# Exogenous melatonin induces drought stress tolerance by promoting plant growth and antioxidant defence system of soybean plants

**DOI:** 10.1093/aobpla/plab026

**Published:** 2021-06-09

**Authors:** Muhammad Imran, Abdul Latif Khan, Raheem Shahzad, Muhammad Aaqil Khan, Saqib Bilal, Adil Khan, Sang-Mo Kang, In-Jung Lee

**Affiliations:** 1 School of Applied Biosciences, Kyungpook National University, 41566Republic of Korea; 2 Natural & Medical Sciences Research Center, University of Nizwa, Nizwa 616, Oman; 3 Department of Horticulture, the University of Haripur, Haripur 21120, Pakistan; 4 Department of Plant and Soil sciences, Texas Tech University, Lubbock, TX 79409, USA

**Keywords:** Antioxidants, drought stress, melatonin, phytohormones, soybean

## Abstract

Melatonin is an indolamine bioactive molecule that regulates a wide range of physiological processes during plant growth and enhances abiotic stress tolerance. Here we examined the putative role of exogenous melatonin application (foliar or root zone) in improving drought stress tolerance in soybean seedlings. Pre-treatment of soybean seedlings with melatonin (50 and 100 µM) was found to significantly mitigate the negative effects of drought stress on plant growth-related parameters and chlorophyll content. The beneficial impacts against drought were more pronounced by melatonin application in the rhizosphere than in foliar treatments. The melatonin-induced enhanced tolerance could be attributed to improved photosynthetic activity, reduction of abscisic acid and drought-induced oxidative damage by lowering the accumulation of reactive oxygen species and malondialdehyde. Interestingly, the contents of jasmonic acid and salicylic acid were significantly higher following melatonin treatment in the root zone than in foliar treatment compared with the control. The activity of major antioxidant enzymes such as superoxide dismutase, catalase, polyphenol oxidase, peroxidase and ascorbate peroxidase was stimulated by melatonin application. In addition, melatonin counteracted the drought-induced increase in proline and sugar content. These findings revealed that modifying the endogenous plant hormone content and antioxidant enzymes by melatonin application improved drought tolerance in soybean seedlings. Our findings provide evidence for the stronger physiological role of melatonin in the root zone than in leaves, which may be useful in the large-scale field level application during drought.

## Teaser

Melatonin is an indolamine bioactive molecule that regulates a wide range of physiological processes during plant growth and enhances abiotic stress tolerance. Here we examined the putative role of exogenous melatonin application (foliar or root zone) in improving drought stress tolerance in soybean seedlings. Pre-treatment of soybean seedlings with melatonin (50 and 100 µM) was found to significantly mitigate the negative effects of drought stress on plant growth-related parameters and chlorophyll content. The beneficial impacts against drought were more pronounced by melatonin application in the rhizosphere than in foliar treatments. The melatonin-induced enhanced tolerance could be attributed to improved photosynthetic activity, reduction of abscisic acid and drought-induced oxidative damage by lowering the accumulation of reactive oxygen species and malondialdehyde. Interestingly, the contents of jasmonic acid and salicylic acid were significantly higher following melatonin treatment in the root zone than following foliar application as compared to the control. The activity of major antioxidant enzymes such as superoxide dismutase, catalase, polyphenol oxidase, peroxidase and ascorbate peroxidase was largely stimulated by melatonin application. In addition, melatonin counteracted the drought-induced increase in proline and sugar content. These findings revealed that modifying the endogenous plant hormone content and antioxidant enzymes by melatonin improved drought tolerance in soybean seedlings. Our findings provide evidence for the stronger physiological role of melatonin in the root zone rather than foliar zone, which may be useful in the large-scale field level application during drought.

## Introduction

In the natural environment, plants are exposed to a wide range of abiotic and biotic stresses such as flooding, drought, heat, salinity, heavy metals and pathogenic microbes, which affect the growth, development and yield of agricultural crops and consequently impact global food security. In this context, drought stress is a crucial problem in arid and semi-arid environments. Drought stress reduces the productivity of crops by impairing physiological and biochemical processes, such as photosynthesis, translocation, respiration, and growth promoters. Drought stress can induce various negative effects on plants at the cellular level by altering the redox homeostasis, which can lead to oxidative damage in plants. Overproduction of ROS such as superoxide anion (O_2_^−^), hydroxyl radical (•OH) and hydrogen peroxide (H_2_O_2_) and the resulting oxidative stress can lead to lipid peroxidation, electron leakage and membrane damage, as well as damage to proteins and nucleic acids, inhibition of the closure of stomata and changes in photosynthetic and enzyme activity (Polle 2001; Asada 2006; Jaspers and Kangasjärvi 2010; Maksup *et al*. 2014). To overcome drought-induced damage, plants activate their defence system by enhancing the accumulation of antioxidant enzymes (ascorbate peroxidase, superoxide dismutase, catalase and guaiacol peroxidase) and non-enzymes such as glutathione, carotenoids, α-tocopherol and ascorbic acid, and activating defence mechanisms involving osmotic adjustment and stomata regulation (Shi *et al*. 2016).

Plants respond to external environmental stimuli such as drought stress via plant growth regulators, including the phytohormone melatonin. Melatonin is an important animal hormone that has been reported to activate various signalling events during plant responses to abiotic and biotic stress conditions, and thus melatonin helps safeguard plants under these stresses (Ahmad *et al*. 2019). Several studies have reported the positive role of melatonin in inducing stress tolerance in plants. For example, melatonin was found to induce tolerance in crops under various abiotic stress conditions such as heavy metals (Posmyk *et al*. 2008), high temperature (Xu 2010; Shi and Chan 2014), salinity (Zhang *et al*. 2014) and leaf senescence (Hernández-Ruiz *et al*. 2005; Tan *et al*. 2013; Zhang *et al*. 2015). During stress, melatonin enhances multiple adaptive responses, such as increasing the stomatal conductance, photosynthetic rate, transpiration rate, mineral uptake, exudation of organic acid anions and phenolic compounds, hormonal regulation, sugar metabolism and ROS scavenging, and regulates the antioxidant enzyme activity that can alleviate oxidative damage to lipids, proteins and nucleic acids (Li *et al*. 2015; Manchester *et al*. 2015; Shi *et al*. 2015b; Ahammed *et al*. 2019). Moreover, it has also been reported that melatonin positively enhanced the assimilation of carbon, increased the chlorophyll level and promoted seed germination by enhancing starch degradation in response to cold stress in wheat plants (Li *et al*. 2018).

Soybean is an important crop and is widely grown, with the total production comprising more than 320 million metric tonnes (Kunert *et al*. 2016). Soybean is susceptible to drought stress (Basal and Szabó 2020), and studies have demonstrated that drought stress causes a significant reduction in seed yield of up to 24–50 % (Hardeland *et al*. 2012; Sadeghipour and Abbasi 2012, Ku *et al*. 2013; Cao *et al*. 2019; Dong *et al*. 2019; Zhang *et al*. 2019). Compared with the benefits of melatonin under various abiotic stress conditions, the underlying mechanism of melatonin in alleviating drought stress has rarely been investigated in crops, especially in soybean. To our knowledge, very few studies have systematically discussed the alleviating role of melatonin in plants grown under drought stress conditions. Specifically, little is known about whether foliar or rhizospheric application of melatonin improves stress tolerance or not. In the current study, we aimed to evaluate the effects of melatonin on growth, antioxidants and phytohormone activity in the amelioration of drought stress in soybean seedlings. The cross-talk between melatonin and soybean stress tolerance mechanisms such as the antioxidant system and phytohormones in response to drought stress is also discussed.

## Materials and Methods

Soybean seeds were provided by the soybean genetic resource centre Kyungpook National University, Daegu, Republic of Korea. Melatonin was purchased from Sigma-Aldrich (CAS NO 73-31-4). The seeds were first surface-sterilized with 2.5 % sodium hypochlorite for 10 min, washed three times with autoclaved distilled water and then germinated. At the VC stage (unifoliate leaf emerged), uniformly germinated seedlings were selected and transferred to plastic pots filled with horticultural substrate containing peat moss (10–15 %), coco peat (45–50 %), perlite (35–40 %) and zeolite (6–8 %) with NO_3_ (∼0.205 mg/g), NH^+^ (∼0.09 mg/g), KO (∼0.1 mg/g) and PO (∼0.35 mg/g) (Khan *et al*. 2019a) and grown in a growth chamber at 24–28 °C, 14/10 h day/night, relative humidity 55–65 % and light intensity 1000 µEm^−2^ s^−1^ from sodium lamps. Plants were exposed to melatonin treatment using foliar spray or root irrigation for 5 days (5 mL of 50 or 100 µM melatonin, twice a day) before exposure to drought stress. Plants grown in normal conditions (without any stress) were supplied with 300–350 mL of water/week and 5 mL of 50 or 100 µM melatonin for 5 days. At the V2 stage (second fully developed trifoliate leaf), the plants were divided into two groups treated as follows: well-watered with foliar and root irrigation, and drought conditions with foliar and root irrigation. The well-watered plants were further divided into: (i) well-watered without any treatment (control), (ii) well-watered plus 50 µM melatonin foliar spray (FM50), (iii) well-watered plus 100 µM melatonin foliar spray (FM100), (iv) well-watered plus 50 µM melatonin root irrigation (RM50) and (v) well-watered plus 100 µM melatonin root irrigation (RM100). The drought-stressed plants were further divided into: (i) drought stress without any treatment (control), (ii) drought plus 50 µM melatonin foliar spray (FM50), (iii) drought plus 100 µM melatonin foliar spray (FM100), (iv) drought plus 50 µM melatonin root irrigation (RM50) and (v) drought plus 100 µM melatonin root irrigation (RM100). Drought was imposed by withholding water until the soil moisture content reached 30–35 % field capacity (FC) for 7 days. Soil moisture content was measured on a daily basis using DEMETRA, E.M. System Soil Tester (Tokyo, Japan). After completion of the stress period, the chlorophyll content of plants was measured using a SPAD 502 (Soil Plant Analysis Development) chlorophyll meter (Kinica Minolta, Tokyo, Japan), after which the plants were harvested, the root and shoot lengths were measured using a scale, then snap-frozen in liquid nitrogen and stored at −80 °C until further analysis.

### Determination of antioxidant enzymatic activity

CAT activity was measured using a previously described method (Halo *et al*. 2015; Bilal *et al*. 2018), which involved calculation of H_2_O_2_ absorption reduction at 240 nm. The reaction buffer contained 15 mM hydrogen peroxide and 50 mM potassium phosphate buffer at a pH of 7.0. Then, 100 μL of the enzyme extract was added to the reaction mixture to initiate the reaction. The H_2_O_2_ level in the reaction mixture was measured after 1 min using the extinction coefficient of 40 mM^−1^ cm^−1^, which indicated CAT enzyme activity.

SOD activity was determined using a previously described method (Giannopolitis and Ries 1977), which consisted of evaluating the ability of SOD to photochemically decrease nitroblue tetrazolium (NBT). SOD activity units were determined as the amount of enzyme required to cause 50 % inhibition of the reduction of NBT, as monitored at 560 nm.

POD and PPO activities were determined using the guaiacol method (Zhang and Kirkham 1994), which was performed by adding 0.1 mL of the supernatant to the reaction mixture containing 1.0 mL of 2 % H_2_O_2_, 2.9 mL of 50 mM phosphate buffer (pH 5.5) and 1.0 mL of 50 mM guaiacol. Phosphate buffer was used as the control without enzyme. Absorbance was read at 470 nm for 3 min, and POD activity was calculated as unit change per minute.

To measure the APX activity, 100 mg of plant sample was extracted with 1 mL of 50 mM phosphate buffer (pH 7.0) containing 1 mM ascorbic acid and 1 mM EDTA. The homogenates were centrifuged at 4830 × g (4 °C) for 15 min. The supernatant was mixed with phosphate buffer solution (pH 7.0), 15 mM ascorbic acid and 0.3 mM H_2_O_2_, and the reaction mixture was read at 290 nm. To determine the reduction in GSH content, a previously described method (Ellman 1959; Asaf *et al*. 2017) was used.

### Relative water content

The relative water concentration (RWC) was measured according to a previously described method (Turk and Erdal 2015). Briefly, the fresh weight (FW) of the sixth leaf was measured after sampling. Leaf segments were immersed overnight in distilled water, and turgid weight (TW) was again measured, after which the leaf segment was oven-dried at 75 °C to measure dry weight. The RWC was calculated using the formula RWC (FW − DW)/(TW − DW) × 100, with three replicates.

### Determination of soluble protein, soluble sugar and proline content

 The soluble protein content was measured as described previously (Ashraf and Iram 2005). Briefly, 0.5 g of fresh leaf sample was homogenized with 10 mL phosphate buffer (pH 7.0) and centrifuged at 10 000 × g for 20 min. SERVA Blue-G was added to the extract, and the absorbance was read at 570 nm using a standard curve (in mg g^−1^/FW): the obtained results were calculated by (C × Vt) = (W × Vs × 100), where C represents the protein content, Vt represents the total volume of the reaction mixture, Vs represents the volume of the supernatant and W represents the weight of the fresh plant sample. Proline content and soluble sugar content were measured using previously described methods (Tiwari *et al*. 2010; Huang *et al*. 2019).

### Extraction and quantification of the phytohormones ABA, SA and JA

Endogenous abscisic acid (ABA) was quantified and extracted as described previously (Qi *et al*. 1998), with slight modifications as reported in recent studies (Khan *et al*. 2020a;Bilal *et al*. 2020). ABA was extracted from the aerial parts of the plant (freeze-dried plant samples, 0.3 g), and a chromatograph was run using the Me-[2H6]-ABA standard. The fraction was methylated with diazomethane for detection, and ABA was quantified using GC-MS (6890N network gas chromatograph, Agilent Technologies). The software from ThermoQuset Corp. (Manchester, UK) was used to monitor signal ions (m/z 162 and 190 for Me-ABA and m/z 166 and 194 for Me-[2H6]-ABA).

Endogenous SA was extracted and quantified using previously described methods (Seskar *et al*. 1998; Khan *et al*. 2020a). Briefly, 0.3 g of a freeze-dried plant shoot was treated with 100 % CH_3_OH (methanol) and centrifuged at 10 000 × g. The methanolic extracts were dried using a vacuum drier. After drying, the pellets were suspended in 2.5 mL of 5 % trichloroacetic acid (TCA) and centrifuged at 10 000 × g. The resulting supernatant was separated using ethyl acetate, cyclopentane and isopropanol (ratio of 100:99:1 v/v) and dried using nitrogen gas, followed by quantification by HPLC (Shimadzu fluorescence detector Shimadzu RF-10AXL) at 305 and 365 nm, equipped with a C18 reverse-phase HPLC column (HP Hypersil ODS, MA, USA). The flow rate was maintained at 1.0 mL min^−1^.

Endogenous JA was extracted and quantified as described previously (Khan *et al*. 2019b, c). Briefly, 0.3 g of frozen and ground plant sample was treated with extraction solution (70:30 v/v acetone and critic acid) and 50 ng of JA standard (9, [10-2H2]-9, 10-dihydro-JA). The extract was allowed to evaporate using a rotary evaporator. The aqueous solution was then filtered/extracted three times using 30 mL diethyl ether. The resulting extraction solution was loaded onto a solid-phase cartridge, and then the cartridge was washed twice with 5 mL of trichloromethane and 2-propanol (2:1 v/v). The bound JA and the standard were washed with 1 mL of diethyl ether/acetic acid (98:2 v/v). Subsequently, the sample was first methylated and then analysed by GCMS (6890N network GC system). The fragment ion was examined at m/z = 83 AMU relative to base peaks of JA and [9, 10-2H2]-9, 10-dihydro-JA.

### Determination of H_2_O_2_, MDA and electrolyte leakage

H_2_O_2_ content was measured using a previously described method (Velikova *et al*. 2000; Yu-Na *et al*. 2020). Briefly, 0.1 g of leaf sample was ground and extracted using 5 mL of 0.1 % TCA and centrifuged at 12 000 × *g* for 15 min. Next, 0.5 mL of the supernatant was collected, and 1 mL of 1 M potassium iodide and 0.5 mL of 10 mM phosphate buffer (pH 7.0) were added, and the absorbance was detected at 390 nm. The H_2_O_2_ content was estimated using the extinction coefficient (ɛ) 0.28 mM cm^−1^ and expressed as μM g^−1^ DW. Lipid peroxidation in leaves was determined by measuring the levels of malondialdehyde (MDA) as described elsewhere (Khan *et al*. 2020c). Briefly, 0.1 g of fresh plant tissue was ground with 10 mL of 5 % TCA and centrifuged at 4000 × g for 10 min at 4 °C. The resulting supernatant was suspended with 4 mL of TBA, heated at 90 °C for 25 min and then immediately cooled down at 4 °C. The sample was centrifuged, and the supernatant was read at wavelengths of 532 and 600 nm. The MDA content was calculated as µmol/g^-1^ of FW. Electrolyte leakage was determined using an electrical conductivity meter (Twin Cond., Horiba, B-17) as described previously (Han *et al*. 2017).

### Statistical analysis

All experiments were performed in triplicate, and data collected from each replicate were pooled together. Mean values are presented with standard error. Significant differences in mean values between each treatment were analysed by one-way analysis of variance, followed by Duncan’s multiple range test using the Statistical Analysis System (SAS 9.1). The GraphPad Prism software (version 6.0, San Diego, CA, USA) was used to describe the results graphically.

## Results

### Melatonin affects key growth factors, biomass and chlorophyll content

**Figure 1.**
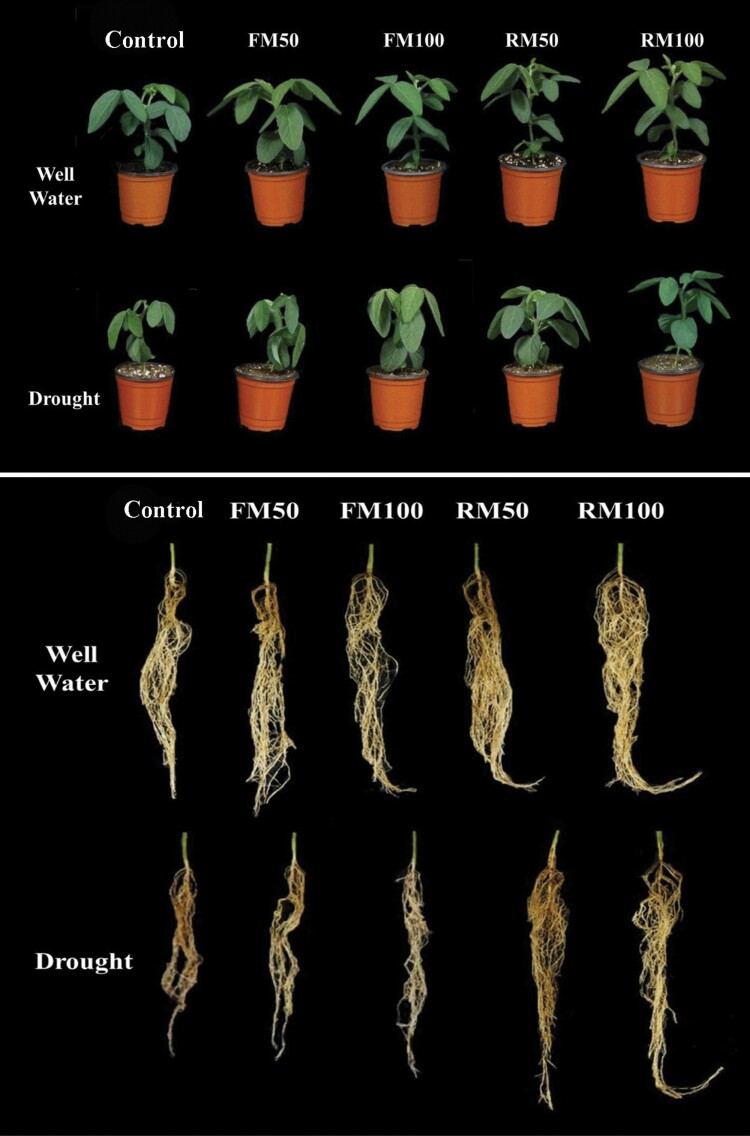
Effects of melatonin application on the phenotypic appearance of soybean plants grown under normal and drought stress conditions.

The results of this study showed that drought stress led to a significant decrease in root/shoot lengths and fresh/dry weights. However, melatonin application had significant effects on plant growth and development. Under normal conditions, melatonin treatment by foliar and root irrigation (FM50, FM100, RM50 and RM100) resulted in a slight increase in root and shoot length, biomass and chlorophyll content compared with control plants. In drought-stressed control plants there was a significant decrease in root length by 46.6 %, shoot length by 34.6 % and fresh and dry weights by 53.1 % and 46.8 %, respectively, compared with those in well-watered control plants. Foliar application of melatonin FM50 resulted in a slight increase in shoot and root length and plant biomass, whereas FM100-treated plants showed significant increases in shoot length (26.78 %), root length (32.7 %), fresh weight (74.1 %), dry weight (70.5 %) and chlorophyll content (19.5 %) compared with drought-stressed control plants in a concentration-dependent manner (Table 1; Fig. 1). Moreover, the application of melatonin via root irrigation (RM50 and RM100) promoted plant growth and development by significantly enhancing the shoot length (44.2 and 55.7 %, respectively), root length (41.4 and 81.1 %, respectively), fresh weight (32.2 and 96.7 %, respectively), dry weight (35.2 and 94.1 %, respectively) and chlorophyll content (20.1 and 36.9 %), respectively, compared with those in plants under drought conditions and treated via foliar application in a concentration-dependent manner (Table 1; Fig. 1). These findings suggest that the application of melatonin via root irrigation has more positive effects on plant growth and development under stress conditions than foliar application, and 100 µM melatonin application can provide more effective protection against drought than 50 µM melatonin. The root irrigation method also resulted in a significantly higher chlorophyll content and plant biomass.

**Table 1.** Effects of exogenous melatonin application on the growth attributes and chlorophyll content of soybean plants with/without drought stress.

Treatment	SL	RL	FW	DW	CHL (SPAD)
Well-water					
Control	20.06 ± 1.6d	15.1 ± 1.7^c^	6.7 ± 0.9b	3.2 ± 0.4c	36.6 ± 0.8c
FM50	21.1 ± 1.7cd	16.06 ± 1.02c	6.7 ± 1.0b	3.4 ± 0.4c	36.8 ± 0.6c
FM100	23.4 ± 1.4bc	16.3 ± 1.0bc	8.6 ± 0.9ab	4.8 ± 0.5b	39.1 ± 1.4b
RM50	25.06 ± 2.0ab	18.6 ± 1.1b	8.7 ± 1.05ab	4.9 ± 0.6b	36.8 ± 0.9c
RM100	26.7 ± 1.5a	21.2 ± 1.3a	10.7 ± 1.7a	6.7 ± 0.5a	42.3 ± 1.4a
Drought					
Control	13.1 ± 1.9d	8.06 ± 1.3c	3.1 ± 0.6c	1.7 ± 0.3c	26.8 ± 1.9c
FM50	14.01 ± 1.0cd	8.4 ± 1.3c	3.3 ± 0.6c	1.7 ± 0.4c	29.03 ± 1.9c
FM100	16.6 ± 1.5bc	10.7 ± 1.2b	5.4 ± 0.7ab	2.9 ± 0.2ab	32.03 ± 1.9b
RM50	18.9 ± 1.5ab	11.4 ± 1.5b	4.3 ± 0.6bc	2.3 ± 0.2b	33.1 ± 1.3b
RM100	20.4 ± 1.6a	14.6 ± 2.0a	6.1 ± 0.5a	3.3 ± 0.2a	36.7 ± 1.6a

Control (well-watered and drought stress without any treatment,) FM50/FM100 (50 µM/100 µM foliar Melatonin application well-watered and Drought), RM50/RM100 (50 µM/100 µM root-irrigated Melatonin application well-watered and Drought). Shoot length (SL/cm), Root length (RL/cm), Fresh weight (FW/g), Dry weight (DW/g) and Chlorophyll content (CHL/SPAD). Each data point is the mean of at least three replicates. Mean (± SE) followed by the different letter (s) are significantly different from each other as evaluated by DMRT.

### Melatonin activates antioxidant enzymes

**Figure 2.**
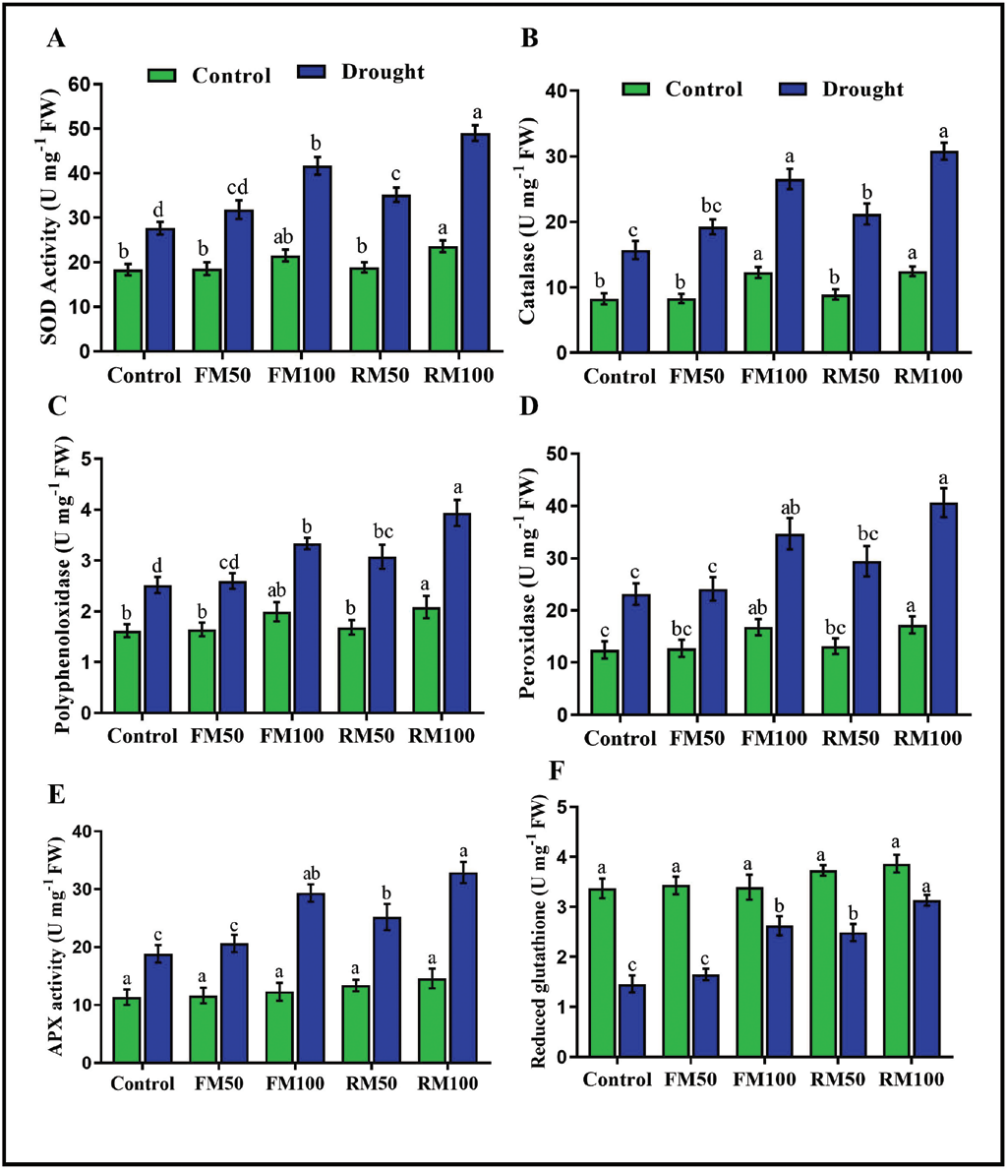
Effects of exogenous application of melatonin on antioxidant enzyme activity: (A) Superoxide dismutase, (B) Catalase, (C) Polyphenoloxidase, (D) Peroxidase, (E) Ascorbate peroxidase and (F) GSH content in soybean plants under normal and drought stress conditions. Each data point represents mean ± SD (*n* = 3). Bars with different letters are significantly different from each other, as evaluated by DMRT.

Melatonin functions as an antioxidant enzyme activator and protects plants from oxidative damage. In the present study, the effect of drought stress on antioxidant enzyme (SOD, CAT, PPO, POD, APX and GSH) activity in soybean plants with or without melatonin treatment was determined. The results showed a significant reduction in SOD, CAT, PPO, POD and APX activity in plants without melatonin treatment compared with melatonin-treated plants under drought conditions. Under normal conditions, melatonin treatment by foliar and root irrigation (FM50, FM100, RM50 and RM100) resulted in a slight increase in antioxidant enzyme activity. Similarly, plants treated with foliar application of melatonin (FM50) showed a slight increase in SOD and CAT activity, and FM100-treated plants showed significant increases in SOD (50.5 %) and CAT (68.7 %) activity compared with control plants under drought conditions. Moreover, root irrigation with melatonin (RM50 and RM100) significantly enhanced SOD (27.07 and 77.07 %, respectively) and CAT (35.03 and 96.1 %, respectively) activity compared with that in drought-treated control plants (Fig. 2A and B). The activity of POD and PPO did not differ in plants treated with FM50 and drought-treated control plants under the stress condition, whereas plants treated with FM100 showed significant increases in POD and PPO activity by 50.2 and 31.9 %, respectively, compared with drought-treated control plants. Root irrigation with melatonin was more efficient than foliar application of melatonin, as it increased the activity of POD and PPO by 27.2 and 22.7 %, respectively, in plants treated with RM50, and by 75.7 and 55.9 %, respectively, in plants treated with RM100 compared with drought-treated control plants in a concentration-dependent manner (Fig. 2C and D). In addition, melatonin application resulted in significant increases in the activity of APX and GSH in FM100- (by 55.8 and 85.7 %, respectively) and in RM100-treated plants (by 74.9 and 96.3 %, respectively) compared with drought-treated control plants. The maximum APX and GSH activity was observed in plants treated with FM100 and RM100, with higher levels than other treatments (Fig. 2E and F). This result suggests that 100 µM melatonin application by root irrigation was the most efficient at inducing drought stress tolerance in plants and protecting them from oxidative damage.

### Effects of melatonin on H_2_O_2_, MDA and electrolyte leakage accumulation

**Figure 3.**
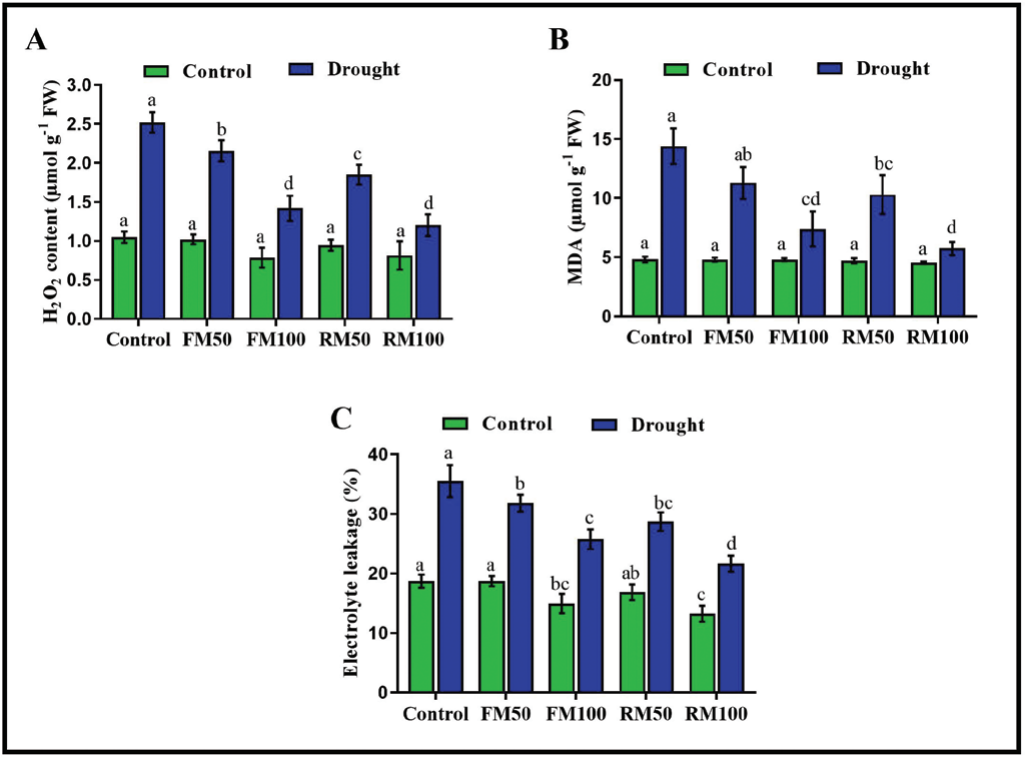
Effects of exogenous application of melatonin on (A) H_2_O_2,_ (B) Malondialdehyde and (C) Electrolyte leakage in soybean plants under normal and drought stress conditions. Each data point represents mean ± SD (*n* = 3). Bars with different letters are significantly different from each other, as evaluated by DMRT.

Hydrogen peroxide is produced by cellular metabolism, and is an indicator of the ROS scavenging capacity of plants under stress, wherein melatonin is a highly effective ROS scavenger. The results of this study show that H_2_O_2_ production in soybean plants significantly increased in drought-stressed control plants compared with well-watered control plants. Foliar application of melatonin (FM50 and FM100) reduced the accumulation of H_2_O_2_ by 15.9 and 44.0 %, respectively, compared with drought-stressed control plants, whereas plants that were root-irrigated with melatonin (RM50 and RM100) showed a more effective reduction of H_2_O_2_ accumulation, by 27.9 and 52.0 %, respectively, compared with drought-treated control plants (Fig. 3A).

To evaluate the effects of drought stress and melatonin treatment on membrane integrity, MDA levels and electrolyte leakage were investigated. The results showed that drought stress significantly enhanced the MDA content and electrolyte leakage, whereas in plants subjected to foliar and root irrigation with melatonin (FM50, FM100, RM50 and RM100), there was a significant decrease in MDA content by 21.6, 48.9, 27.9 and 60.1 %, respectively, and in electrolyte leakage by 10.4, 27.6, 19.1 and 39.1 %, respectively, compared with drought-stressed control plants (Fig. 3B and C). Furthermore, root irrigation with melatonin resulted in a more effective reduction in MDA content and electrolyte leakage than foliar application. These findings suggest that root irrigation with 100 µM melatonin was more effective at scavenging ROS accumulation than foliar application.

### Relative water content

**Figure 4.**
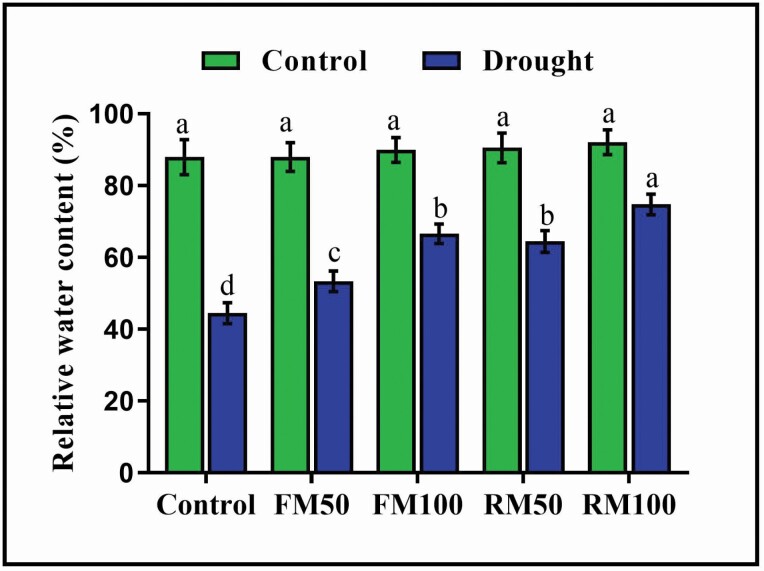
Effects of exogenous application of melatonin on relative water content (RWC) in soybean plants under normal and drought stress conditions. Each data point represents mean ± SD (*n* = 3). Bars with different letters are significantly different from each other, as evaluated by DMRT.

Relative water content (RWC) is the primary factor indicating the water status and ability of plants to survive under stress conditions. The results of this study showed a significant decrease (49.4 %) in RWC during drought stress in drought-treated control soybean plants compared to that in well-watered control plants. In contrast, plants treated with melatonin via foliar and root irrigation showed a significant improvement in RWC under the drought stress (Fig. 4). The most effective increase in RWC was observed in FM100 and RM100, with the increase in RWC values being 49.9 and 68.2 %, respectively, compared with those in drought-treated control plants (Fig. 4). Compared with foliar application, root irrigation with 100 µM melatonin led to a greater increase in RWC.

### Melatonin improves phytohormone contents

**Figure 5.**
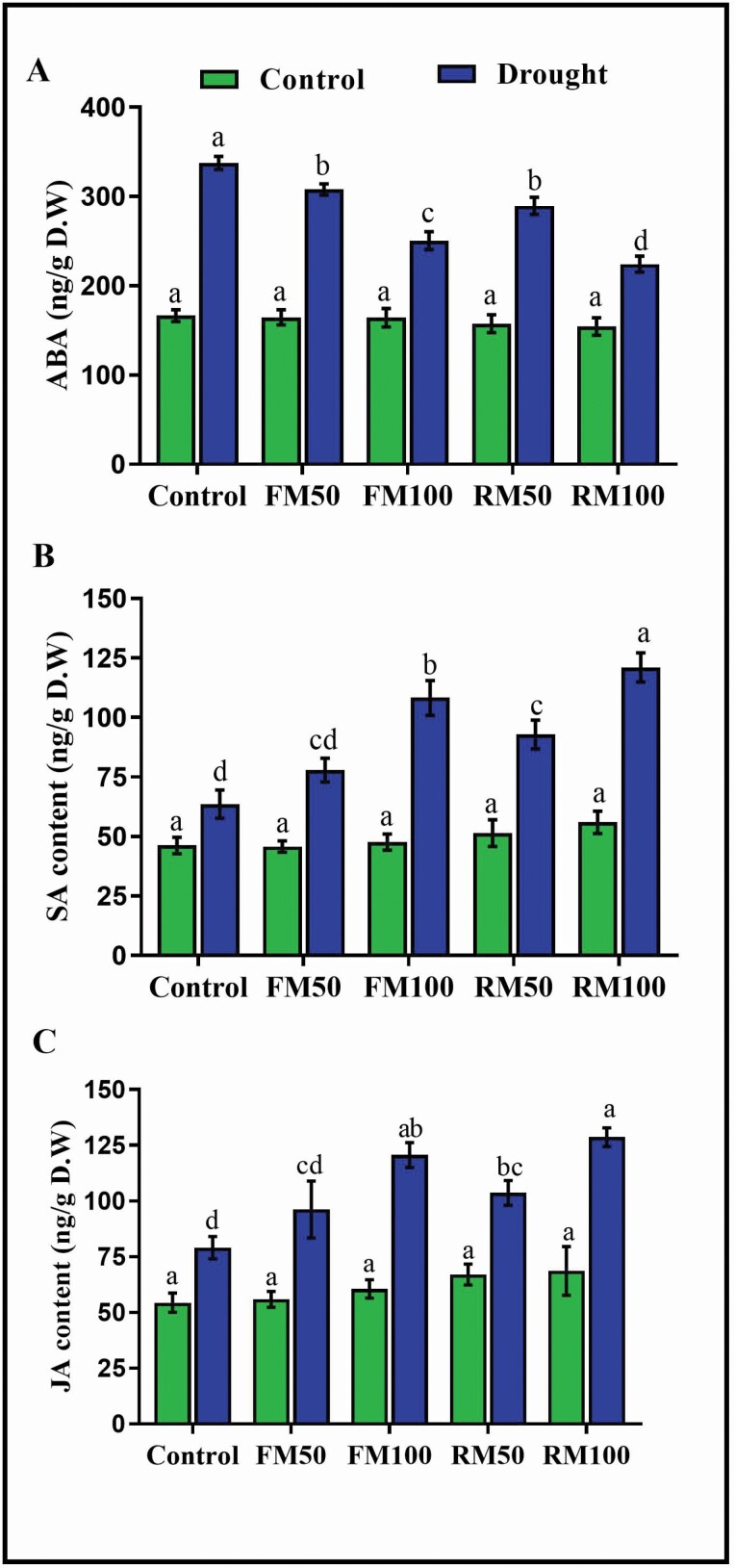
Effects of melatonin application on the plant phytohormones: (A) Abscisic acid, (B) Salicylic acid and (C) Jasmonic acid in soybean plants under normal and drought stress conditions. Each data point represents mean ± SD (*n* = 3). Bars with different letters are significantly different from each other, as evaluated by DMRT.

We investigated the possible role of melatonin in response to phytohormones during drought stress. The results showed that under normal conditions, melatonin did not induce abscisic acid (ABA) accumulation, with no significant difference compared with the well-watered control plants, whereas in the drought-treated control plants, there was a significant increase in ABA accumulation by 102.7 % compared with that in well-watered control plants. Furthermore, plants treated with foliar application of melatonin (FM50 and FM100) showed a slight decrease (8.7 and 25.7 %, respectively) in ABA accumulation compared with drought-treated control plants. The root irrigation method was more effective at reducing ABA production; plants treated with RM50 and RM100 showed a significant reduction (14.2 and 33.6 %, respectively) in ABA production compared to drought-treated control plants (Fig. 5A). Salicylic acid (SA) also has a well-established physiological role in plant growth and development. The results of the present study showed that under normal conditions, melatonin treatment had no significant effect on SA content compared with well-watered control plants. However, under stress conditions, a slight difference was observed in SA content in FM50- and RM50-treated plants, whereas the FM100- and RM100-treated plants showed a significantly higher accumulation of SA, by 70.1 and 90.3 %, respectively, compared with that in drought-treated control plants (Fig. 5B). Similarly, exogenous melatonin application by both foliar spray (FM100) and root irrigation (RM100) significantly increased the accumulation of jasmonic acid (JA) by 52.5 and 62.6 %, respectively, under the drought conditions (Fig. 5C).

### Effects of melatonin on soluble sugar and proline contents

**Figure 6.**
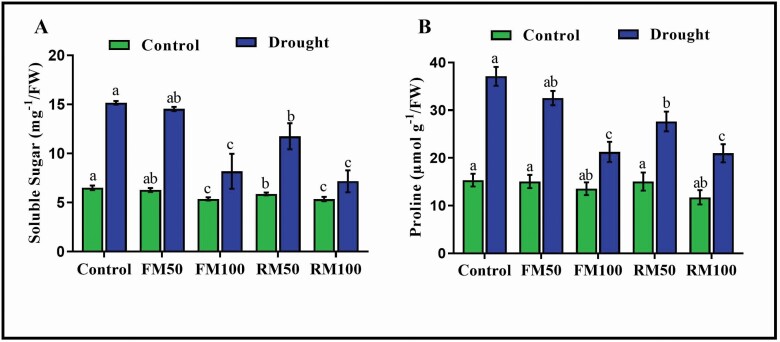
Effects of exogenous application of melatonin on (A) Soluble sugar and (B) Proline in soybean plants under normal and drought stress conditions. Each data point represents mean ± SD (*n* = 3). Bars with different letters are significantly different from each other, as evaluated by DMRT.

Proline and soluble sugar are compatible solutes that can balance water potential, improve cytoplasmic osmotic pressure, protect the membrane system and thereby reduce water loss from cells. The results of the present study showed that drought stress considerably increased the soluble sugar and proline contents, which led to biochemical alterations in soybean plants. Moreover, the soluble sugar and proline contents were significantly decreased in melatonin-treated plants to a variable degree. Compared with drought-treated control plants, FM50-treated plants showed no significant differences and RM50-treated plants showed slight decreases in sugar and proline contents by 22.5 and 25.6 %, respectively. In contrast, plants treated with FM100 showed significant decreases in soluble sugar and proline contents by 46.3 and 42.8 %, respectively, compared with drought-treated control plants. Moreover, the application of melatonin by root irrigation (RM100) caused a significant reduction of 52.9 % in soluble sugar content and 43.6 % in proline content compared with drought-treated control plants (Fig. 6). These results suggest that the soluble sugar, protein and proline in the shoots of soybean plants adopt different strategies to avoid drought stress, and that exogenous melatonin application may reverse these changes, with the root irrigation method having a better protective effect than the foliar application method.

## Discussion

Drought is the one of the major environmental factors that can restrict plant growth and development; it can also cause a wide range of physiological, anatomical and morphological effects in plants (Liu *et al.* 2015). In the present study drought stress reduced soybean plant growth and development by reducing the efficiency of photosynthesis and plant biomass and increasing ROS accumulation and the MDA level, while melatonin application increased the soybean growth parameters and chlorophyll content in plants exposed to drought stress (Table 1; Fig. 1). Similarly Han *et al*. (2017) reported that exogenous melatonin application significantly improved the growth and development of rice seedlings, and enhanced the rate of photosynthesis and photosystem II activity by enhancing the antioxidant enzyme activity, which alleviated the accumulation of ROS and MDA induced in cells by cold stress. Under stress conditions, the chlorophyll content was significantly decreased, whereas melatonin application effectively inhibited its degradation and increased the chlorophyll content to improve plant photosynthesis (Shi *et al*. 2015c). Chlorophyll is involved in photosynthesis and plays an essential role in the transmission and absorption of light energy (Arnao and Hernández-Ruiz 2009). The results of this study indicate that the application of an optimum concentration of melatonin significantly enhanced the biosynthesis of chlorophyll and increased the plant root and shoot length and biomass. We also found that the root irrigation method of melatonin application was more effective than the foliar application method and reduced the effects induced by drought stress in soybean plants.

Under stress conditions, plants generate more ROS that lead to the induction of peroxidation of membrane lipids and oxidative damage (Kar 2011), and in response to this oxidative damage, plants evolve tolerance mechanisms to maintain cell homeostasis and resist the abiotic stress (Yildiztugay *et al*. 2017; Khan *et al*. 2020b), and activate several enzymes, including POD, CAT, APX and SOD to protect against oxidative damage. The balance of these antioxidant enzymes is the key to inhibiting the production of ROS (Yildiztugay *et al*. 2017). Furthermore SOD plays a vital role in scavenging ROS and converting the O_2_^-^ to O_2_ and H_2_O_2,_ after which CAT and POD break down the H_2_O_2_ into water molecules (Hu *et al*. 2016). Accordingly, the present study showed that drought-stressed plants exhibited a decline in the activity of antioxidant enzymes, whereas plants treated with 100 µM by foliar or root irrigation showed significant increases in the activity of SOD, CAT, PPO, POD and APX and inhibition of H_2_O_2_ accumulation under stress conditions. In contrast, control soybean plants exposed to drought stress showed increased accumulation of H_2_O_2_ and decreased activity of CAT, PPO, POD, APX and SOD (Fig. 2). Similarly Li *et al*. (2015) reported that during drought stress plants increase the H_2_O_2_ and electrolyte leakage levels whereas melatonin pre-treated plants reduced the H_2_O_2_ and electrolyte leakage levels and increased antioxidant enzyme activity. The reduction in H_2_O_2_ content might be related to the antioxidant enzyme activity in melatonin-treated plants. The major function of melatonin, along with its effects on antioxidant enzyme activity, may be to maintain intracellular H_2_O_2_ accumulation at a steady level (Cui *et al*. 2017). Moreover, 100 µm exogenous melatonin alleviated ROS accumulation and cold-induced oxidative damage through the scavenging of ROS and enhancing the activity of antioxidants in bermuda grass (Shi *et al*. 2015a). In the current study, 100 µM exogenous melatonin application clearly alleviated oxidative damage in soybean leaves, especially when using the root irrigation method, which suggests that under drought stress, exogenous melatonin treatment effectively protected the cell membrane against oxidative damage. Moreover, the different methods of foliar spray or root irrigation at different concentrations resulted in different patterns of increased antioxidant enzyme activity.

Melatonin is a hydrophilic and lipophilic molecule that can distribute in the cytoplasm and lipid membranes and locate in the hydrophilic side of the lipid bilayer (Catalá 2007; Huang *et al*. 2019). Melatonin molecules arrange themselves parallel to the lipid tail at low concentrations and parallel to the bilayer at high concentrations, which suggests that the organization of melatonin in lipid membranes is dependent on its concentration (Huang *et al*. 2019). Han *et al*. (2017) reported significant increases in lipid peroxidation during cold stress. Melatonin scavenges ROS in plants under drought stress and can protect the cell wall; this fact was supported by the present study results, wherein there was a reduction in MDA level, electrolyte leakage and H_2_O_2_ accumulation in melatonin-treated plants (Fig. 3). Furthermore Li *et al*. (2018) reported that 100 µm melatonin enhanced plants’ tolerance to cold, drought and salt by reducing the ROS burst, maintaining photosynthetic efficiency, decreasing the MDA level and enhancing antioxidant activity in tea plants. H_2_O_2_ is directly involved in the regulation of stomatal movement, and thus the opening and closing of stomata represent the physiological responses of plants to drought stress (Savchenko *et al*. 2014). The accumulation of ABA in plant cells is associated with the formation of ROS. With lower production of endogenous ABA, the accumulation of H_2_O_2_ is also reduced. Therefore, the relationship between ABA and H_2_O_2_ plays an important role during drought stress (Liu *et al*. 2010; Ye *et al*. 2011). The high production of ABA can promote ROS formation and cause oxidative damage, such as leaf peroxidation, electrolyte leakage, *Chl* degradation and reduced photosynthetic performance (Jiang and Zhang 2002). Moreover melatonin application induced the expression of the ABA catabolism gene CYP707 and downregulated the ABA biosynthesis gene NCED, resulting in decreased ABA levels (Zhang *et al*. 2014). This was confirmed in the present study where, under normal conditions, exogenous application of melatonin did not alter ABA accumulation, whereas under stress conditions, melatonin application suppressed the accumulation of ABA (Fig. 5). Similar results were reported by (Li *et al*. 2015), who found that melatonin application caused a reduction in the ABA content through the regulation of ABA catabolism and biosynthesis genes.

Jasmonic acid and salicylic acid, the plant signalling and growth-regulating molecules, also play a significant role in resistance to abiotic and biotic stress. JA plays a vital role in abiotic stress tolerance by enhancing photosynthetic efficiency and antioxidant metabolism (Rincón-Pérez *et al*. 2016). JA is involved in responses such as stomatal regulation, activation of the antioxidant system and accumulation of amino acids and soluble sugar (Wagner *et al*. 2013). In the present study, melatonin application significantly enhanced the JA level, thus minimizing water loss. Similarly, Savchenko *et al*. (2014) reported that JA regulates the stomata and controls water loss. SA is a phenolic compound that has a great potential to influence seed germination, root initiation, transpiration and photosynthesis (Verma and Agrawal 2017). Kareem *et al*. (2019) reported that SA enhanced wheat biomass and upregulated the CBF14 gene, which improved the wheat plants’ biomass grain weight under drought stress. Similarly Sharma *et al*. (2020) reported that melatonin positively upregulated JA accumulation during drought stress. Melatonin also enhances the SA level in response to drought stress. SA regulates the plant’s physiological response and plays an important role in response to environmental conditions such as drought (Khan *et al*. 2012), cold (Ilyas *et al*. 2017) and salinity (Ndamukong *et al*. 2007). It was reported that in arabidopsis pathogen infection was induced at higher melatonin and SA levels, while the SNAT (*N*-acetyltransferase) knockout mutant caused a reduction in the melatonin and SA levels (Li *et al*. 2015). During stress conditions melatonin increased the SA and NO levels in tobacco plants (Zhao *et al*. 2019). Although SA and melatonin have been demonstrated to have an important role in plants’ physiological and molecular responses to biotic and abiotic stresses and share the same biosynthesis pathway and precursor (chorismic acid), these regulatory molecules have not been directly compared (Hernández-Ruiz and Arnao 2018; Albacete 2020). However, our results suggest that melatonin interacts with other plant hormones to tolerate stress and plays a vital role in plant defence against abiotic stress.

Drought stress may cause the instability of cell membranes, and osmotic regulation is the basic response to this stress (Huang *et al*. 2019). Therefore, the levels of the two osmotic regulators proline and soluble sugar often increase under stress conditions (Huang *et al*. 2019), as confirmed in the present study, wherein exogenous melatonin application substantially reduced the accumulation of proline and soluble sugar under the drought stress condition, and 100 µM melatonin application by root irrigation was more effective than foliar application (Fig. 6). Based on the results of this study and those of previous studies, it can be stated that during drought stress, exogenous melatonin application promotes plant growth and development, activates the antioxidant enzymes and reduces ROS accumulation; moreover, the mitigation potential of exogenous melatonin application is related to its concentration based on the application method.

## Conclusion

This study demonstrated that the melatonin-induced improvement in drought stress tolerance in soybean plants was associated with enhanced functioning of the antioxidant defence machinery and the scavenging of H_2_O_2_, which alleviated the oxidative impairment induced by drought stress. Exogenous application of melatonin, especially at 100 µM concentration, significantly improved the growth of soybean plants by inhibiting membrane injury and reducing H_2_O_2_ concentrations under moderate and severe drought stress. These effects are probably achieved by regulating the activity of the antioxidant enzymes SOD, CAT, GPX and APX. Melatonin reduced oxidative damage and improved water status, enabling the plants to maintain a higher total chlorophyll content. In addition, the possible cross-talk between melatonin and phytohormones may play a crucial role in drought stress tolerance in soybean plants, providing additional insights into melatonin signal transduction in plants subjected to drought stress, although the complex molecular system operating during drought stress enabled by melatonin needs to be further examined in detail. Furthermore, the root zone application of melatonin resulted in significantly higher physiological and phytohormonal regulation than foliar application. This could be an essential factor determining melatonin application at large-scale field levels. The findings of this study provide evidence for the physiological role of melatonin and serve as a platform for its possible application in agricultural or related fields of research.

## Notes

**Phenome, Genome & Environment. Chief Editor:** Colleen Doherty

## References

[CIT0001] Ahammed GJ , Xu W, Liu A, Chen S. 2019. Endogenous melatonin deficiency aggravates high temperature-induced oxidative stress in *Solanum lycopersicum* L. Environmental and Experimental Botany 161:303–311.

[CIT0002] Ahmad S , Kamran M, Ding R, Meng X, Wang H, Ahmad I, Fahad S, Han Q. 2019. Exogenous melatonin confers drought stress by promoting plant growth, photosynthetic capacity and antioxidant defense system of maize seedlings. PeerJ 7:e7793. 31616591 10.7717/peerj.7793 PMC6791350

[CIT0003] Albacete A . 2020. Get together: the interaction between melatonin and salicylic acid as a strategy to improve plant stress tolerance. Murcia: Multidisciplinary Digital Publishing Institute.

[CIT0004] Arnao MB , Hernández-Ruiz J. 2009. Protective effect of melatonin against chlorophyll degradation during the senescence of barley leaves. Journal of Pineal Research 46:58–63. 18691358 10.1111/j.1600-079X.2008.00625.x

[CIT0005] Asada K . 2006. Production and scavenging of reactive oxygen species in chloroplasts and their functions. Plant Physiology 141:391–396. 16760493 10.1104/pp.106.082040 PMC1475469

[CIT0006] Asaf S , Khan AL, Khan MA, Imran QM, Yun BW, Lee IJ. 2017. Osmoprotective functions conferred to soybean plants via inoculation with *Sphingomonas* sp. LK11 and exogenous trehalose. Microbiological Research 205:135–145. 28942839 10.1016/j.micres.2017.08.009

[CIT0007] Ashraf M , Iram A. 2005. Drought stress induced changes in some organic substances in nodules and other plant parts of two potential legumes differing in salt tolerance. Flora-Morphology, Distribution, Functional Ecology of Plants 200:535–546.

[CIT0008] Basal O , Szabó A. 2020. Physiomorphology of soybean as affected by drought stress and nitrogen application. Scientifica 2020:6093836. 32351758 10.1155/2020/6093836 PMC7171675

[CIT0009] Bilal S , Khan AL, Shahzad R, Kim YH, Imran M, Khan MJ, Al-Harrasi A, Kim TH, Lee IJ. 2018. Mechanisms of Cr(VI) resistance by endophytic *Sphingomonas* sp. LK11 and its Cr(VI) phytotoxic mitigating effects in soybean (*Glycine max* L.). Ecotoxicology and Environmental Safety 164:648–658. 30170313 10.1016/j.ecoenv.2018.08.043

[CIT0010] Bilal S , Shahzad R, Imran M, Jan R, Kim KM, Lee I-J. 2020. Synergistic association of endophytic fungi enhances *Glycine max* L. resilience to combined abiotic stresses: heavy metals, high temperature and drought stress. Industrial Crops and Products 143:111931.

[CIT0011] Cao L , Jin X, Zhang Y. 2019. Melatonin confers drought stress tolerance in soybean (*Glycine max* L.) by modulating photosynthesis, osmolytes, and reactive oxygen metabolism. Photosynthetica 57:812–819.

[CIT0012] Catalá A . 2007. The ability of melatonin to counteract lipid peroxidation in biological membranes. Current Molecular Medicine 7:638–649. 18045142 10.2174/156652407782564444

[CIT0013] Cui G , Zhao X, Liu S, Sun F, Zhang C, Xi Y. 2017. Beneficial effects of melatonin in overcoming drought stress in wheat seedlings. Plant Physiology and Biochemistry 118:138–149. 28633086 10.1016/j.plaphy.2017.06.014

[CIT0014] Dong S , Jiang Y, Dong Y, Wang L, Wang W, Ma Z, Yan C, Ma C, Liu L. 2019. A study on soybean responses to drought stress and rehydration. Saudi Journal of Biological Sciences 26:2006–2017. 31889786 10.1016/j.sjbs.2019.08.005 PMC6923469

[CIT0015] Ellman M . 1959. A spectrophotometric method for determination of reduced glutathione in tissues. Analytical Biochemistry 74:214–226. 10.1016/0003-2697(76)90326-2 962076

[CIT0016] Giannopolitis CN , Ries SK. 1977. Superoxide dismutases: I. Occurrence in higher plants. Plant Physiology 59:309–314. 16659839 10.1104/pp.59.2.309 PMC542387

[CIT0017] Halo BA , Khan AL, Waqas M, Al-Harrasi A, Hussain J, Ali L, Adnan M, Lee I-J. 2015. Endophytic bacteria (*Sphingomonas* sp. LK11) and gibberellin can improve *Solanum lycopersicum* growth and oxidative stress under salinity. Journal of Plant Interactions 10:117–125.

[CIT0018] Han QH , Huang B, Ding CB, Zhang ZW, Chen YE, Hu C, Zhou LJ, Huang Y, Liao JQ, Yuan S, Yuan M. 2017. Effects of melatonin on anti-oxidative systems and photosystem II in cold-stressed rice seedlings. Frontiers in Plant Science 8:785. 28553310 10.3389/fpls.2017.00785 PMC5425610

[CIT0019] Hardeland R , Madrid JA, Tan DX, Reiter RJ. 2012. Melatonin, the circadian multioscillator system and health: the need for detailed analyses of peripheral melatonin signaling. Journal of Pineal Research 52:139–166. 22034907 10.1111/j.1600-079X.2011.00934.x

[CIT0020] Hernández-Ruiz J , Arnao MB. 2018. Relationship of melatonin and salicylic acid in biotic/abiotic plant stress responses. Agronomy 8:33.

[CIT0021] Hernández-Ruiz J , Cano A, Arnao MB. 2005. Melatonin acts as a growth-stimulating compound in some monocot species. Journal of Pineal Research 39:137–142. 16098090 10.1111/j.1600-079X.2005.00226.x

[CIT0022] Hu Z , Fan J, Xie Y, Amombo E, Liu A, Gitau MM, Khaldun ABM, Chen L, Fu J. 2016. Comparative photosynthetic and metabolic analyses reveal mechanism of improved cold stress tolerance in bermudagrass by exogenous melatonin. Plant Physiology and Biochemistry 100:94–104. 26807934 10.1016/j.plaphy.2016.01.008

[CIT0023] Huang B , Chen Y-E, Zhao Y-Q, Ding C-B, Liao J-Q, Hu C, Zhou L-J, Zhang Z-W, Yuan S, Yuan M. 2019. Exogenous melatonin alleviates oxidative damages and protects photosystem ii in maize seedlings under drought stress. Frontiers in Plant Science 10:677. 10.3389/fpls.2019.00677 PMC6543012 31178885

[CIT0024] Ilyas N , Gull R, Mazhar R, Saeed M, Kanwal S, Shabir S, Bibi F. 2017. Influence of salicylic acid and jasmonic acid on wheat under drought stress. Communications in Soil Science and Plant Analysis 48:2715–2723.

[CIT0025] Jaspers P , Kangasjärvi J. 2010. Reactive oxygen species in abiotic stress signaling. Physiologia Plantarum 138:405–413. 20028478 10.1111/j.1399-3054.2009.01321.x

[CIT0026] Jiang M , Zhang J. 2002. Water stress-induced abscisic acid accumulation triggers the increased generation of reactive oxygen species and up-regulates the activities of antioxidant enzymes in maize leaves. Journal of Experimental Botany 53:2401–2410. 12432032 10.1093/jxb/erf090

[CIT0027] Kar RK . 2011. Plant responses to water stress: role of reactive oxygen species. Plant Signaling & Behavior 6:1741–1745. 22057331 10.4161/psb.6.11.17729 PMC3329347

[CIT0028] Kareem F , Rihan H, Fuller MP. 2019. The effect of exogenous applications of salicylic acid on drought tolerance and up-regulation of the drought response regulon of Iraqi wheat. Journal of Crop Science and Biotechnology 22:37–45.

[CIT0029] Khan NA , Nazar R, Iqbal N, Anjum NA. 2012. Phytohormones and abiotic stress tolerance in plants. Berlin and Heidelberg: Springer Science & Business Media.

[CIT0030] Khan MA , Asaf S, Khan AL, Adhikari A, Jan R, Ali S, Imran M, Kim KM, Lee IJ. 2019a. Halotolerant rhizobacterial strains mitigate the adverse effects of NaCl stress in soybean seedlings. Biomed Research International 2019:15. 10.1155/2019/9530963 PMC6925695 31886270

[CIT0031] Khan MA , Asaf S, Khan AL, Ullah I, Ali S, Kang S-M, Lee I-J. 2019b. Alleviation of salt stress response in soybean plants with the endophytic bacterial isolate *Curtobacterium* sp. SAK1. Annals of Microbiology 69:797–808.

[CIT0032] Khan MA , Ullah I, Waqas M, Hamayun M, Khan AL, Asaf S, Kang S-M, Kim K-M, Jan R, Lee I-J. 2019c. Halo-tolerant rhizospheric *Arthrobacter woluwensis* AK1 mitigates salt stress and induces physio-hormonal changes and expression of GmST1 and GmLAX3 in soybean. Symbiosis 77:9–21.

[CIT0033] Khan A , Khan AL, Imran M, Asaf S, Kim Y-H, Bilal S, Numan M, Al-Harrasi A, Al-Rawahi A, Lee I-J. 2020a. Silicon-induced thermotolerance in *Solanum lycopersicum* L. via activation of antioxidant system, heat shock proteins, and endogenous phytohormones. BMC Plant Biology 20:1–18. 32493420 10.1186/s12870-020-02456-7 PMC7268409

[CIT0034] Khan A , Numan M, Khan AL, Lee I-J, Imran M, Asaf S, Al-Harrasi A. 2020b. Melatonin: awakening the defense mechanisms during plant oxidative stress. Plants 9:407. 10.3390/plants9040407 PMC7238205 32218185

[CIT0035] Khan MA , Asaf S, Khan AL, Jan R, Kang SM, Kim KM, Lee IJ. 2020c. Extending thermotolerance to tomato seedlings by inoculation with SA1 isolate of Bacillus cereus and comparison with exogenous humic acid application. PLoS One 15:e0232228. 32353077 10.1371/journal.pone.0232228 PMC7192560

[CIT0072] Kim Y-N, Khan MA, Kang S-M, Hamayun M, Lee I-J. 2020. Enhancement of drought-stress tolerance of *Brassica oleracea* var. *italica* L. by newly isolated *Variovorax *sp. YN_A59_. Journal of Microbiology and Biotechnology 30:1500–1509. 10.4014/jmb.2006.06010 PMC9728237 32807757

[CIT0036] Ku Y-S , Au-Yeung W-K, Yung Y-L, Li M-W, Wen C-Q, Liu X, Lam H-M. 2013. Drought stress and tolerance in soybean. A comprehensive survey of international soybean research—Genetics, physiology, agronomy and nitrogen relationships 2:209–237.

[CIT0037] Kunert KJ , Vorster BJ, Fenta BA, Kibido T, Dionisio G, Foyer CH. 2016. Drought stress responses in soybean roots and nodules. Frontiers in Plant Science 7:1015. 10.3389/fpls.2016.01015 PMC4941547 27462339

[CIT0038] Li X , Brestic M, Tan DX, Zivcak M, Zhu X, Liu S, Song F, Reiter RJ, Liu F. 2018. Melatonin alleviates low PS I-limited carbon assimilation under elevated CO2 and enhances the cold tolerance of offspring in chlorophyll b-deficient mutant wheat. Journal of Pineal Research 64:e12453. 10.1111/jpi.12453 29149482

[CIT0039] Li C , Tan DX, Liang D, Chang C, Jia D, Ma F. 2015. Melatonin mediates the regulation of ABA metabolism, free-radical scavenging, and stomatal behaviour in two *Malus* species under drought stress. Journal of Experimental Botany 66:669–680. 25481689 10.1093/jxb/eru476

[CIT0040] Liu J , Wang W, Wang L, Sun Y. 2015. Exogenous melatonin improves seedling health index and drought tolerance in tomato. Plant Growth Regulation 77:317–326.

[CIT0041] Liu Y , Ye N, Liu R, Chen M, Zhang J. 2010. H2O2 mediates the regulation of ABA catabolism and GA biosynthesis in *Arabidopsis* seed dormancy and germination. Journal of Experimental Botany 61:2979–2990. 20460363 10.1093/jxb/erq125 PMC2892143

[CIT0042] Maksup S , Roytrakul S, Supaibulwatana K. 2014. Physiological and comparative proteomic analyses of Thai jasmine rice and two check cultivars in response to drought stress. Journal of Plant Interactions 9:43–55.

[CIT0043] Manchester LC , Coto-Montes A, Boga JA, Andersen LP, Zhou Z, Galano A, Vriend J, Tan DX, Reiter RJ. 2015. Melatonin: an ancient molecule that makes oxygen metabolically tolerable. Journal of Pineal Research 59:403–419. 26272235 10.1111/jpi.12267

[CIT0044] Ndamukong I , Abdallat AA, Thurow C, Fode B, Zander M, Weigel R, Gatz C. 2007. SA-inducible *Arabidopsis* glutaredoxin interacts with TGA factors and suppresses JA-responsive PDF1. 2 transcription. The Plant Journal 50:128–139. 17397508 10.1111/j.1365-313X.2007.03039.x

[CIT0045] Polle A . 2001. Dissecting the superoxide dismutase-ascorbate-glutathione-pathway in chloroplasts by metabolic modeling. Computer simulations as a step towards flux analysis. Plant Physiology 126:445–462. 11351106 10.1104/pp.126.1.445 PMC102317

[CIT0046] Posmyk MM , Kuran H, Marciniak K, Janas KM. 2008. Presowing seed treatment with melatonin protects red cabbage seedlings against toxic copper ion concentrations. Journal of Pineal Research 45:24–31. 18205729 10.1111/j.1600-079X.2007.00552.x

[CIT0047] Qi Q , Rose PA, Abrams GD, Taylor DC, Abrams SR, Cutler AJ. 1998. (+)-Abscisic acid metabolism, 3-ketoacyl-coenzyme A synthase gene expression, and very-long-chain monounsaturated fatty acid biosynthesis in *Brassica napus* embryos. Plant Physiology 117:979–987. 9662540 10.1104/pp.117.3.979 PMC34952

[CIT0048] Rincón-Pérez J , Rodríguez-Hernández L, Ruíz-Valdiviezo VM, Abud-Archila M, Luján-Hidalgo MC, Ruiz-Lau N, González-Mendoza D, Gutiérrez-Miceli FA. 2016. Fatty acids profile, phenolic compounds and antioxidant capacity in elicited callus of *Thevetia peruviana* (Pers.) K. Schum. Journal of Oleo Science 65:311–318. 26972464 10.5650/jos.ess15254

[CIT0073] Sadeghipour O, Abbasi S. 2012. Soybean response to drought and seed inoculation. World Applied Sciences Journal 17:55–60.

[CIT0049] Savchenko T , Kolla VA, Wang CQ, Nasafi Z, Hicks DR, Phadungchob B, Chehab WE, Brandizzi F, Froehlich J, Dehesh K. 2014. Functional convergence of oxylipin and abscisic acid pathways controls stomatal closure in response to drought. Plant Physiology 164:1151–1160. 24429214 10.1104/pp.113.234310 PMC3938610

[CIT0050] Seskar M , Shulaev V, Raskin I. 1998. Endogenous methyl salicylate in pathogen-inoculated tobacco plants. Plant Physiology 116:387–392.

[CIT0051] Sharma A , Wang J, Xu D, Tao S, Chong S, Yan D, Li Z, Yuan H, Zheng B. 2020. Melatonin regulates the functional components of photosynthesis, antioxidant system, gene expression, and metabolic pathways to induce drought resistance in grafted *Carya cathayensis* plants. The Science of the Total Environment 713:136675. 32019031 10.1016/j.scitotenv.2020.136675

[CIT0052] Shi H , Chan Z. 2014. The cysteine2/histidine2-type transcription factor ZINC FINGER OF ARABIDOPSIS THALIANA 6-activated C-REPEAT-BINDING FACTOR pathway is essential for melatonin-mediated freezing stress resistance in Arabidopsis. Journal of Pineal Research 57:185–191. 24962049 10.1111/jpi.12155

[CIT0053] Shi H , Chen K, Wei Y, He C. 2016. Fundamental issues of melatonin-mediated stress signaling in plants. Frontiers in Plant Science 7:1124. 27512404 10.3389/fpls.2016.01124 PMC4961697

[CIT0054] Shi H , Jiang C, Ye T, Tan DX, Reiter RJ, Zhang H, Liu R, Chan Z. 2015a. Comparative physiological, metabolomic, and transcriptomic analyses reveal mechanisms of improved abiotic stress resistance in bermudagrass [*Cynodon dactylon* (L). Pers.] by exogenous melatonin. Journal of Experimental Botany 66:681–694. 25225478 10.1093/jxb/eru373 PMC4321537

[CIT0055] Shi H , Qian Y, Tan DX, Reiter RJ, He C. 2015b. Melatonin induces the transcripts of CBF/DREB1s and their involvement in both abiotic and biotic stresses in *Arabidopsis*. Journal of Pineal Research 59:334–342. 26182834 10.1111/jpi.12262

[CIT0056] Shi H , Reiter RJ, Tan DX, Chan Z. 2015c. INDOLE-3-ACETIC ACID INDUCIBLE 17 positively modulates natural leaf senescence through melatonin-mediated pathway in *Arabidopsis*. Journal of Pineal Research 58:26–33. 25324183 10.1111/jpi.12188

[CIT0057] Tan DX , Manchester LC, Liu X, Rosales-Corral SA, Acuna-Castroviejo D, Reiter RJ. 2013. Mitochondria and chloroplasts as the original sites of melatonin synthesis: a hypothesis related to melatonin’s primary function and evolution in eukaryotes. Journal of Pineal Research 54:127–138. 23137057 10.1111/jpi.12026

[CIT0058] Tiwari JK , Munshi AD, Kumar R, Pandey RN, Arora A, Bhat JS, Sureja AK. 2010. Effect of salt stress on cucumber: Na+–K+ ratio, osmolyte concentration, phenols and chlorophyll content. Acta Physiologiae Plantarum 32:103–114.

[CIT0059] Turk H , Erdal S. 2015. Melatonin alleviates cold-induced oxidative damage in maize seedlings by up-regulating mineral elements and enhancing antioxidant activity. Journal of Plant Nutrition and Soil Science 178:433–439.

[CIT0060] Velikova V , Yordanov I, Edreva A. 2000. Oxidative stress and some antioxidant systems in acid rain-treated bean plants: protective role of exogenous polyamines. Plant Science 151:59–66.

[CIT0061] Verma K , Agrawal S. 2017. Salicylic acid-mediated defence signalling in respect to its perception, alteration and transduction. In: Salicylic acid: a multifaceted hormone. Singapore: Springer, 97–122.

[CIT0062] Wagner A , Tobimatsu Y, Goeminne G, Phillips L, Flint H, Steward D, Torr K, Donaldson L, Boerjan W, Ralph J. 2013. Suppression of CCR impacts metabolite profile and cell wall composition in *Pinus radiata* tracheary elements. Plant Molecular Biology 81:105–117. 23131896 10.1007/s11103-012-9985-z

[CIT0063] Xu X . 2010. Effects of exogenous melatonin on physiological response of cucumber seedlings under high temperature stress. Master’s degree thesis, Northwest A and F University. 21328946

[CIT0064] Ye N , Zhu G, Liu Y, Li Y, Zhang J. 2011. ABA controls H2O2 accumulation through the induction of OsCATB in rice leaves under water stress. Plant and Cell Physiology 52:689–698. 21398647 10.1093/pcp/pcr028

[CIT0065] Yildiztugay E , Ozfidan-Konakci C, Kucukoduk M, Tekis SA. 2017. The impact of selenium application on enzymatic and non-enzymatic antioxidant systems in *Zea mays* roots treated with combined osmotic and heat stress. Archives of Agronomy and Soil Science 63:261–275.

[CIT0067] Zhang M , He S, Zhan Y, Qin B, Jin X, Wang M, Zhang Y, Hu G, Teng Z, Wu Y. 2019. Exogenous melatonin reduces the inhibitory effect of osmotic stress on photosynthesis in soybean. PLoS One 14:e0226542. 31869357 10.1371/journal.pone.0226542 PMC6927616

[CIT0068] Zhang J , Kirkham MB. 1994. Drought-stress-induced changes in activities of superoxide dismutase, catalase, and peroxidase in wheat species. Plant and Cell Physiology 35:785–791.

[CIT0069] Zhang N , Sun Q, Zhang H, Cao Y, Weeda S, Ren S, Guo YD. 2015. Roles of melatonin in abiotic stress resistance in plants. Journal of Experimental Botany 66:647–656. 25124318 10.1093/jxb/eru336

[CIT0070] Zhang HJ , Zhang N, Yang RC, Wang L, Sun QQ, Li DB, Cao YY, Weeda S, Zhao B, Ren S, Guo YD. 2014. Melatonin promotes seed germination under high salinity by regulating antioxidant systems, ABA and GA_4_ interaction in cucumber (*Cucumis sativus* L.). Journal of Pineal Research 57:269–279. 25112973 10.1111/jpi.12167

[CIT0071] Zhao L , Chen L, Gu P, Zhan X, Zhang Y, Hou C, Wu Z, Wu YF, Wang QC. 2019. Exogenous application of melatonin improves plant resistance to virus infection. Plant Pathology 68:1287–1295.

